# Maternal Exposure to Iodine Excess Throughout Pregnancy and Lactation Induces Hypothyroidism in Adult Male Rat Offspring

**DOI:** 10.1038/s41598-017-15529-9

**Published:** 2017-11-15

**Authors:** Caroline Serrano-Nascimento, Rafael Barrera Salgueiro, Thiago Pantaleão, Vânia Maria Corrêa da Costa, Maria Tereza Nunes

**Affiliations:** 1 Department of Physiology and Biophysics, Institute of Biomedical Sciences, University of São Paulo, São Paulo, Brazil; 2 Carlos Chagas Filho Biophysics Institute, Federal University of Rio de Janeiro, Rio de Janeiro, Brazil

## Abstract

This study aimed to investigate the consequences of maternal exposure to iodine excess (IE; 0.6 mg NaI/L) throughout pregnancy and lactation on the hypothalamus-pituitary-thyroid axis of the male offspring in adulthood. Maternal IE exposure increased hypothalamic *Trh* mRNA expression and pituitary Tsh expression and secretion in the adult male offspring. Moreover, the IE-exposed offspring rats presented reduced thyroid hormones levels, morphological alterations in the thyroid follicles, increased thyroid oxidative stress and decreased expression of thyroid differentiation markers (Tshr, Nis, Tg, Tpo, Mct8) and thyroid transcription factors (Nkx2.1, Pax8). Finally, the data presented here strongly suggest that epigenetic mechanisms, as increased DNA methylation, augmented DNA methyltransferases expression, hypermethylation of histone H3, hypoaceylation of histones H3 and H4, increased expression/activity of histone deacetylases and decreased expression/activity of histone acetyltransferases are involved in the repression of thyroid gene expression in the adult male offspring. In conclusion, our results indicate that rat dams’ exposure to IE during pregnancy and lactation induces primary hypothyroidism and triggers several epigenetic changes in the thyroid gland of their male offspring in adulthood.

## Introduction

Thyroxin (T_4_) and triiodothyronine (T_3_) are the main hormones produced by the thyroid gland and are essential for growth, development and metabolism control in vertebrates^1^. Thyroid hormones (TH) are the only hormones that contain iodine, which is essential both for TH synthesis and for thyroid function control^2^. Indeed, iodide uptake is mediated by the activity of the sodium-iodide symporter (NIS) and is the first and limiting step for TH synthesis^3^.

Iodine is scarce in the environment and its consumption through natural foods as well as through iodinated salt supposedly guarantee an adequate supply of this trace element for TH synthesis^4^. According to the World Health Organization (WHO), the iodine consumption should be 100–150 µg per day to guarantee adequate TH production in children and adults^5^. Nevertheless, during specific periods as pregnancy and lactation, WHO suggests a consumption of 200–250 µg iodine per day^6^. Importantly, adequate iodine consumption during these critical periods of development guarantees normal maternal and foetal thyroid function.

The deleterious effects of iodine deficiency (ID) to maternal thyroid function and to foetus development are clearly reported^7^. However, the effects of maternal ingestion of iodine excess (IE) on foetal development are still controversial. In fact, the safety of upper limits of iodine intake is not completely defined^8^. Recent studies have shown that IE consumption during pregnancy and/or lactation impaired maternal thyroid function as well as promoted deleterious effects on the offspring development^9–12^. Additionally, previous studies have shown that hormonal/nutritional disturbances during pregnancy and lactation may interfere with the programming of gene expression through epigenetic mechanisms, as DNA methylation, histones post-translational modifications and differential miRNA expression^13–15^.

Although previous studies have reported the development of goitre in the foetus of mothers exposed to high iodine intake^16,17^, the effects of IE on the programming of thyroid dysfunction in the offspring during adulthood have never been described. Therefore, this study aimed to investigate the consequences of rat dams’ exposure to IE throughout pregnancy and lactation on the hypothalamus-pituitary-thyroid axis function of their male offspring in adult life. In addition, this study also intended to elucidate whether maternal IE exposure induces epigenetic changes in the thyroid of the male offspring.

## Results

### Altered morphometry parameters and serum hormonal levels in the adult male offspring of IE-exposed rat dams

As shown in Table 1, maternal IE exposure during pregnancy and lactation has not altered the body weight (BW) of the adult male rat offspring. Conversely, these animals presented a significant reduction of the dry heart weight (DHW) and the ratio between dry heart weight and body weight (DHW/BW) in comparison to the control group. Interestingly, maternal IE ingestion reduced the male offspring’s circulating levels of T_3_ and T_4_, and significantly increased the serum TSH levels in these animals in adulthood.

**Table 1:** Body weight (BW), dry heart weight (DHW), dry heart to body weight ratio (DHW/BW), T_3_, T_4_ and TSH serum concentrations of adult male offspring of rat dams exposed or not to IE treatment during pregnancy and lactation periods.

	Control	5-HI
*BW* (*g*)	354 ± 11.5	336 ± 6.4
*DHW* (*g*)	0.27 ± 0.01	0.22 ± 0.01**
*DHW/BW* (*g*)^A^	0.76 ± 0.02	0.66 ± 0.01**
*T*_*3*_ (ng/dL)	25.0 ± 1.1	18.4 ± 0.7**
T_4_ (μg/dL)	7.06 ± 0.2	6.31 ± 0.2*
TSH (ng/mL)	1.2 ± 0.1	3.3 ± 0.5***

^A^Values multiplied by 1000. Results are expressed by means ± SEM, *n* = 6-10 per group. *P < 0.05, **P < 0.01, ***P < 0.01 vs. Control.

### Altered hypothalamic and pituitary gene expression in the adult male offspring of IE-exposed rat dams

**Figure 1:**
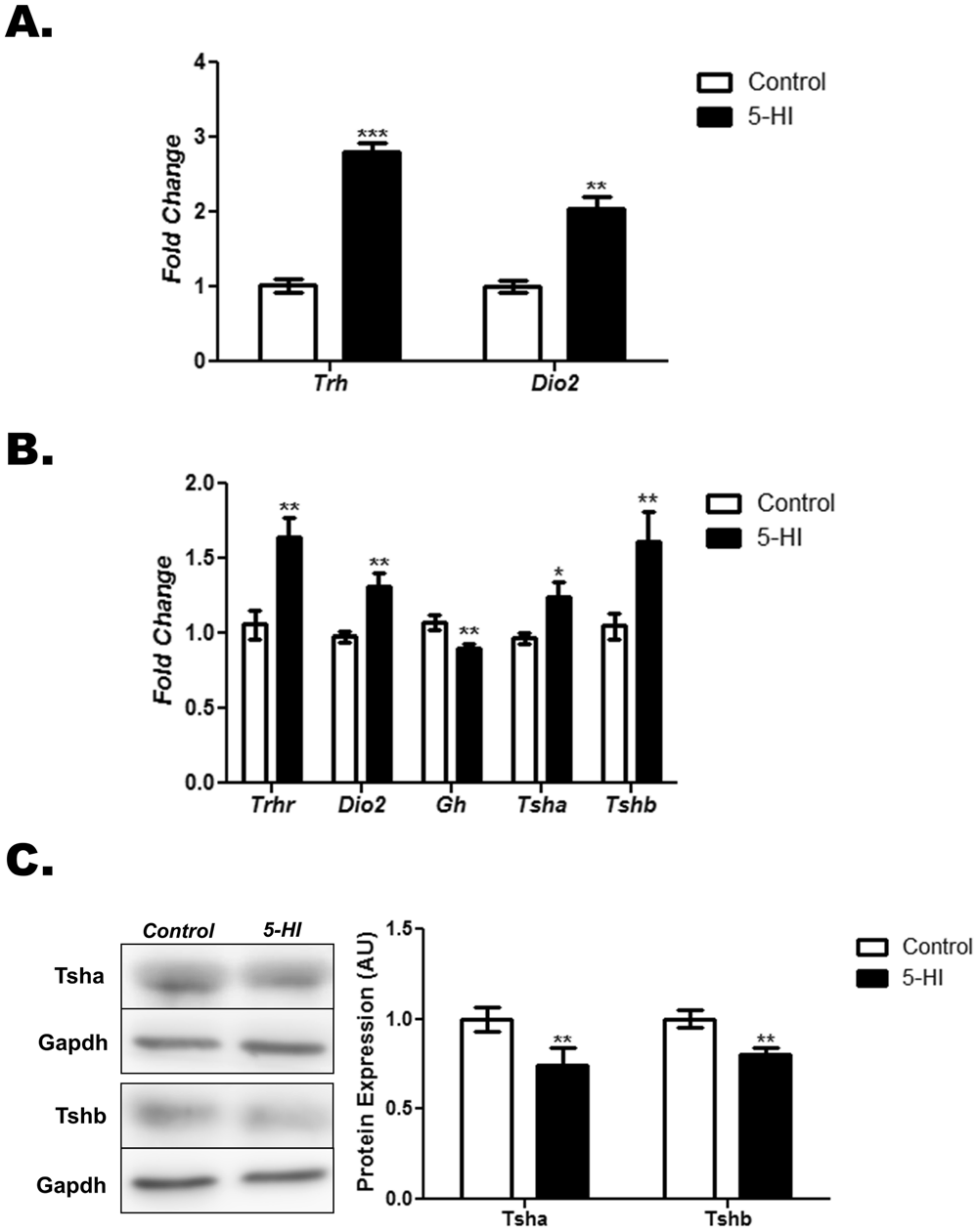
Maternal IE exposure alters gene expression in the hypothalamus and the pituitary of the adult male offspring. Hypothalamus and pituitary gene and/or protein expression were investigated in the adult male offspring of control (C) or IE-exposed rat dams (5-HI). Hypothalamic expression of *Trh* and *Dio2* mRNAs (**A**) as well as pituitary *Trhr*, *Dio2, Gh, Tsha*, *Tshb* mRNA content (**B**) were analyzed by Real-Time PCR and normalized to *Rpl19* mRNA content (n = 10/group). (**C**) Total pituitary content of Tsha and Tshb subunits were analyzed through Western blotting, using Gapdh as loading control (n = 10/group). Representative western blots are shown in the left panel. Results are expressed as means ± SEM as fold change or in arbitrary units (AU). *P < 0.05, **P < 0.01; ***P < 0.001 vs. C (*Unpaired two tailed Student’s t-test*).

At PND90, the IE-exposed male offspring presented higher expression of the *Trh* and *Dio2* mRNAs in the hypothalamus in comparison to the control group (Fig. 1A). Moreover, maternal IE treatment during pregnancy and lactation has decreased *Gh* mRNA expression and increased *Trhr, Dio2, Tsha* and *Tshb* mRNA expression in the pituitary of the male rat offspring in adulthood (Fig. 1B). Interestingly, the protein content of both subunits of TSH were significantly reduced in the pituitary of the IE-exposed male offspring (Fig. 1C).

### Maternal IE exposure alters the morphology of the thyroid gland of the adult male offspring

**Figure 2:**
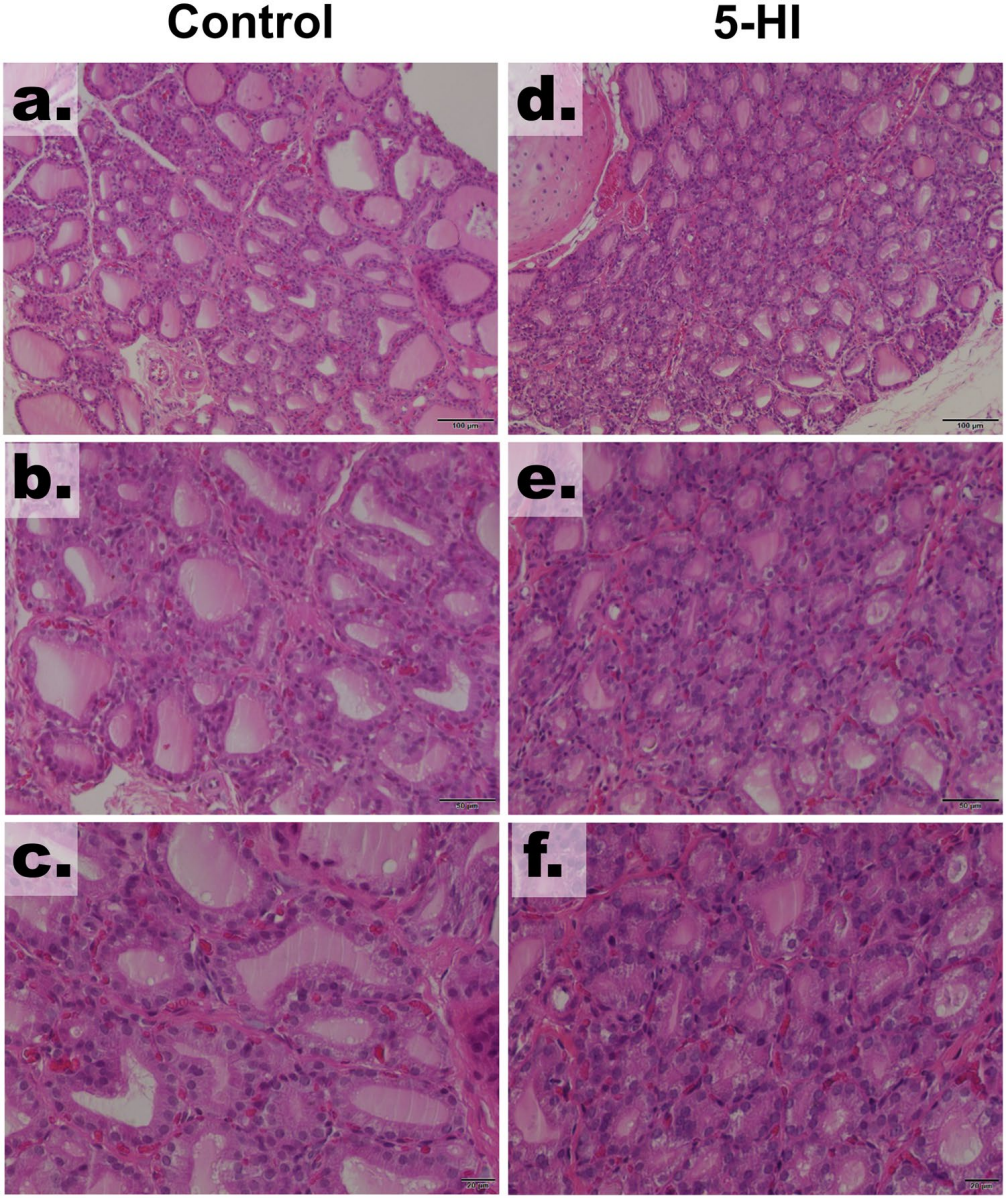
Maternal IE exposure alter the thyroid morphology of the adult male offspring. Light microscope view of the thyroid follicles of the male offspring of control or IE-exposed rat dams. IE treatment decreased the diameter of the thyroid follicles and reduced the amount of colloid within the follicles. No lymphocytic infiltration was observed. Hematoxylin and eosin. Magnification 100× (**a**,**d**), 200× (**b**,**e**) and 400× (**c**,**f**).

As demonstrated in Fig. 2, the male offspring of IE-exposed rat dams presented significant alterations on thyroid histology. Indeed, the offspring of the control dams presented follicles with different size and shape (Fig. 2a–c). In contrast, the adult male offspring of rat dams exposed to the IE treatment (5-HI) presented several follicles with decreased diameter and filled with lower content of thyroglobulin (Fig. 2d–f). No infiltration of immune cells was observed in any of the studied groups.

### Thyroid gene and protein expression in the adult male offspring of IE-exposed rat dams

**Figure 3:**
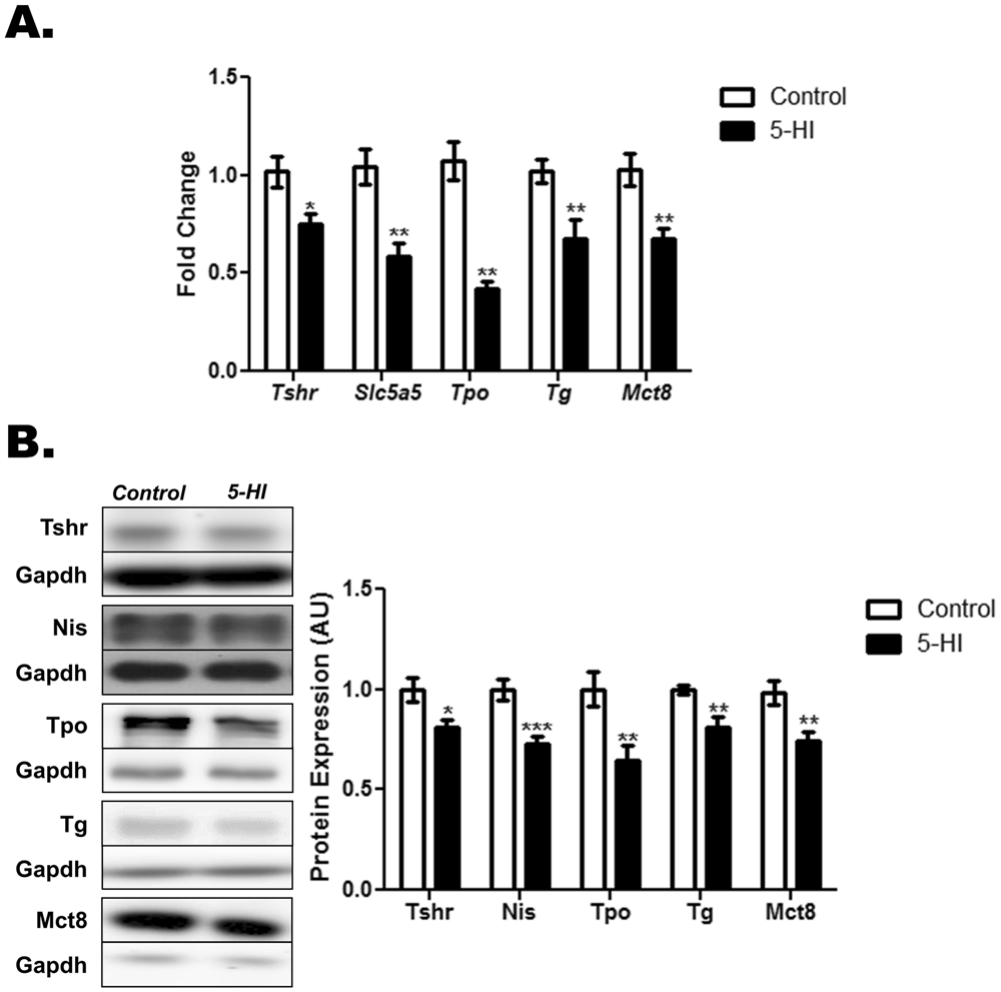
Maternal IE exposure reduces the expression of differentiation genes in the thyroid of the adult male offspring. Thyroid gene and protein expression were investigated in the adult male offspring of control (C) or IE-exposed rat dams (5-HI). (**A**) *Tshr, Slc5a5*, *Tpo, Tg* and *Mct8* mRNA content were analyzed by Real-Time PCR and normalized to *Rpl19* mRNA content (n = 10/group). (**B**) Total thyroid Tshr, Nis, Tpo, Tg and Mct8 protein content were analyzed through Western blotting, using Gapdh as loading control (n = 10/group). Representative western blots are shown in the left panel. Results are expressed as means ± SEM as fold change or arbitrary units (AU). *P < 0.05, **P < 0.01; ***P < 0.001 vs. C (*Unpaired two tailed Student’s t-test*).

**Figure 4:**
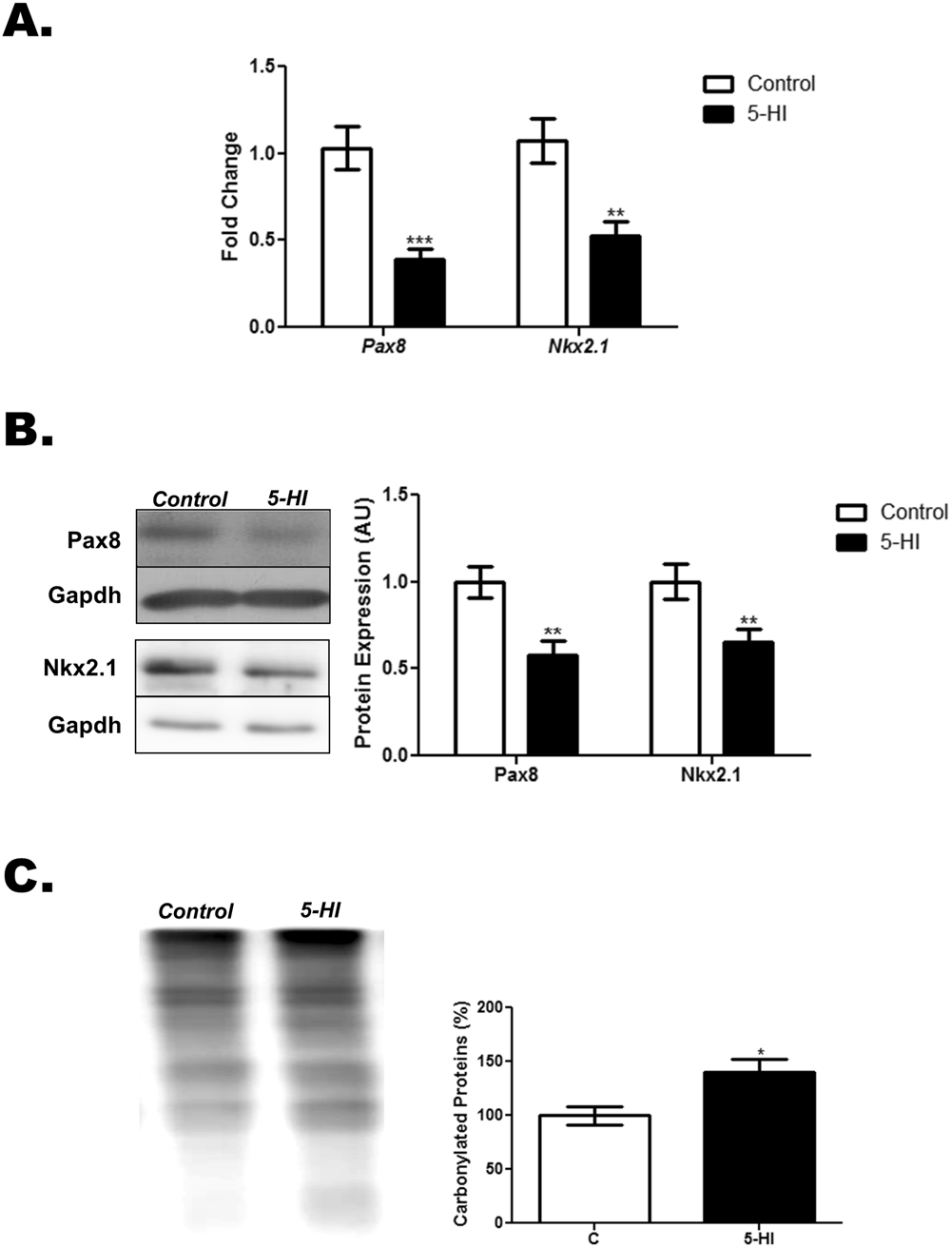
Maternal IE exposure reduces thyroid transcription factors expression and  increases thyroid oxidative stress in the adult male offspring. Thyroid gene expression and protein carbonylation were investigated in the adult male offspring of control (C) or IE-exposed rat dams (5-HI). (**A**) *Pax8* and *Nkx2.1* mRNA content were analyzed by Real-Time PCR and normalized to *Rpl19* mRNA content (n = 10/group). (**B**) Total thyroid Pax8 and Nkx2.1 protein content were analyzed through Western blotting, using Gapdh as loading control (n = 10/group). Representative western blots are shown in the left panel. (**C**) Protein carbonylation was analyzed through Western blotting by using specific primary and secondary antibodies to detect carbonylated proteins. Ponceau S staining was used as loading control (n = 6/group). Representative western blots are shown in the left panel. Results are expressed as means ± SEM as fold change, arbitrary units (AU) or percentage (%), respectively. *P < 0.05, **P < 0.01; ***P < 0.001 vs. C (*Unpaired two tailed Student’s t-test*).

As demonstrated in Figs 3 and 4, maternal exposure to IE during pregnancy and lactation has altered the expression of genes and proteins involved in the TH synthesis and secretion in the thyroid of male rat offspring. Indeed, the thyroid expression of *Tshr, Slc5a5*, *Tpo*, *Tg* and *Mct8* mRNAs (Fig. 3A) and Tshr, Nis, Tpo, Tg and Mct8 proteins (Fig. 3B) were reduced in the IE-exposed male offspring. In agreement, these animals also presented significant reduction of thyroid *Pax8* and *Nkx2.1* mRNA (Fig. 4A) and protein (Fig. 4B) expression.

### Increased thyroid oxidative stress status in the adult male offspring of IE-exposed rat dams

Maternal exposure to IE treatment during pregnancy and lactation has significantly increased the amount of carbonylated proteins in the thyroid of the adult male offspring in comparison to the control animals (Fig. 4C).

### Altered peripheral conversion of TH in the adult male offspring of IE-exposed rat dams

**Figure 5:**
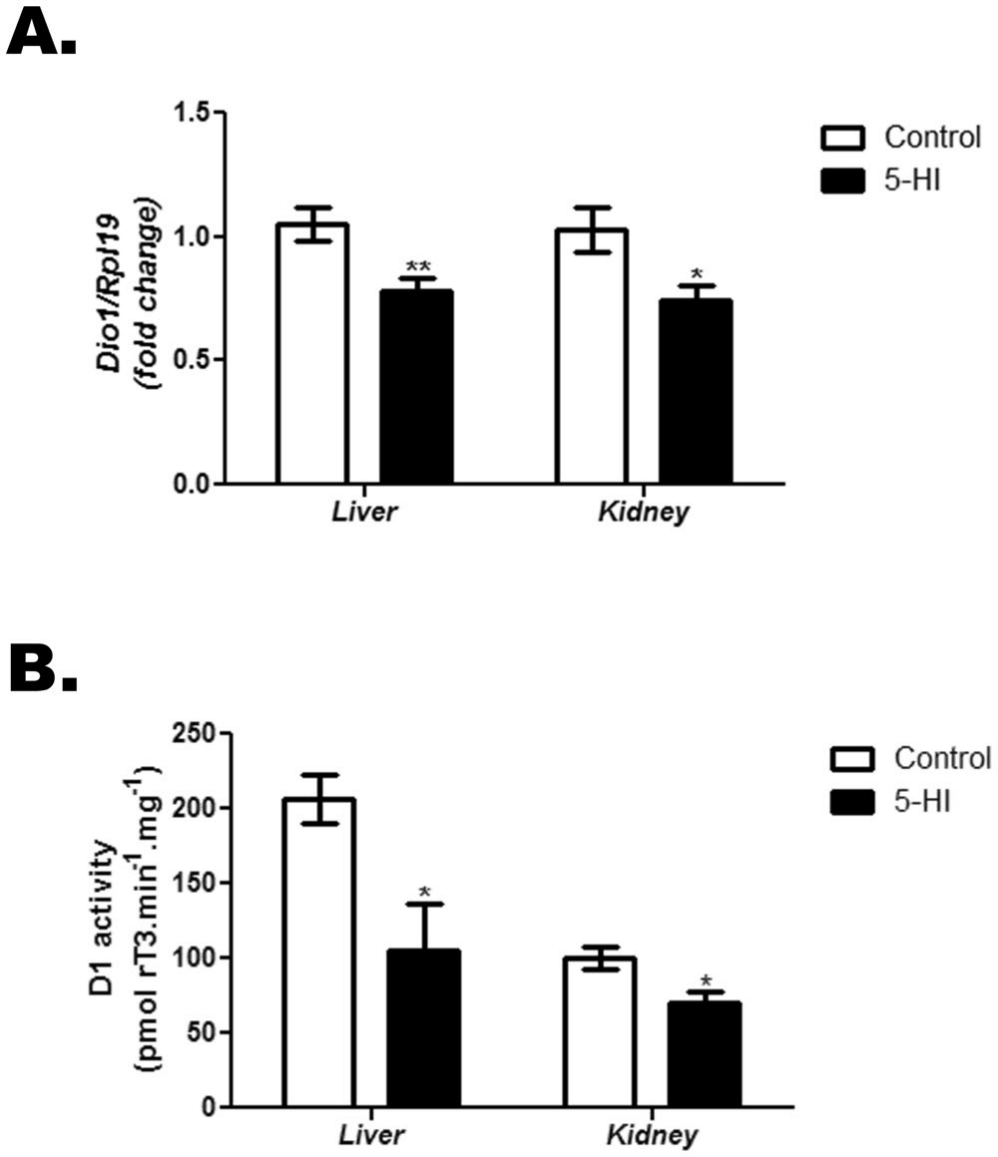
Maternal IE exposure reduces liver and kidney *Dio1* mRNA expression and D1 activity in the adult male offspring. *Dio1* expression and D1 activity were analyzed in the liver and kidney of the adult male offspring of control (C) or IE-exposed rat dams (5-HI). (**A**) *Dio1* mRNA expression was analyzed in the liver and the kidney by Real-Time PCR and normalized to *Rpl19* mRNA content (n = 10/group). (**B**) D1 activity was evaluated in the microsomes from liver and kidney of the offspring rats (n = 3–5/group). Results are expressed as means ± SEM as fold change or as picomols rT3.min^−1^.mg^−1^, respectively. *P < 0.05; ** P < 0.01 vs. C (*Unpaired two tailed Student’s t-test*).

As presented in Fig. 5, maternal IE exposure significantly reduced *Dio1* mRNA expression in the liver and the kidney of the adult male offspring (Fig. 5A). In accordance, the D1 activity was reduced in both tissues of IE-exposed offspring in comparison to control group (Fig. 5B).

### Thyroid DNA methylation status in the adult male offspring of IE-exposed rat dams

**Figure 6:**
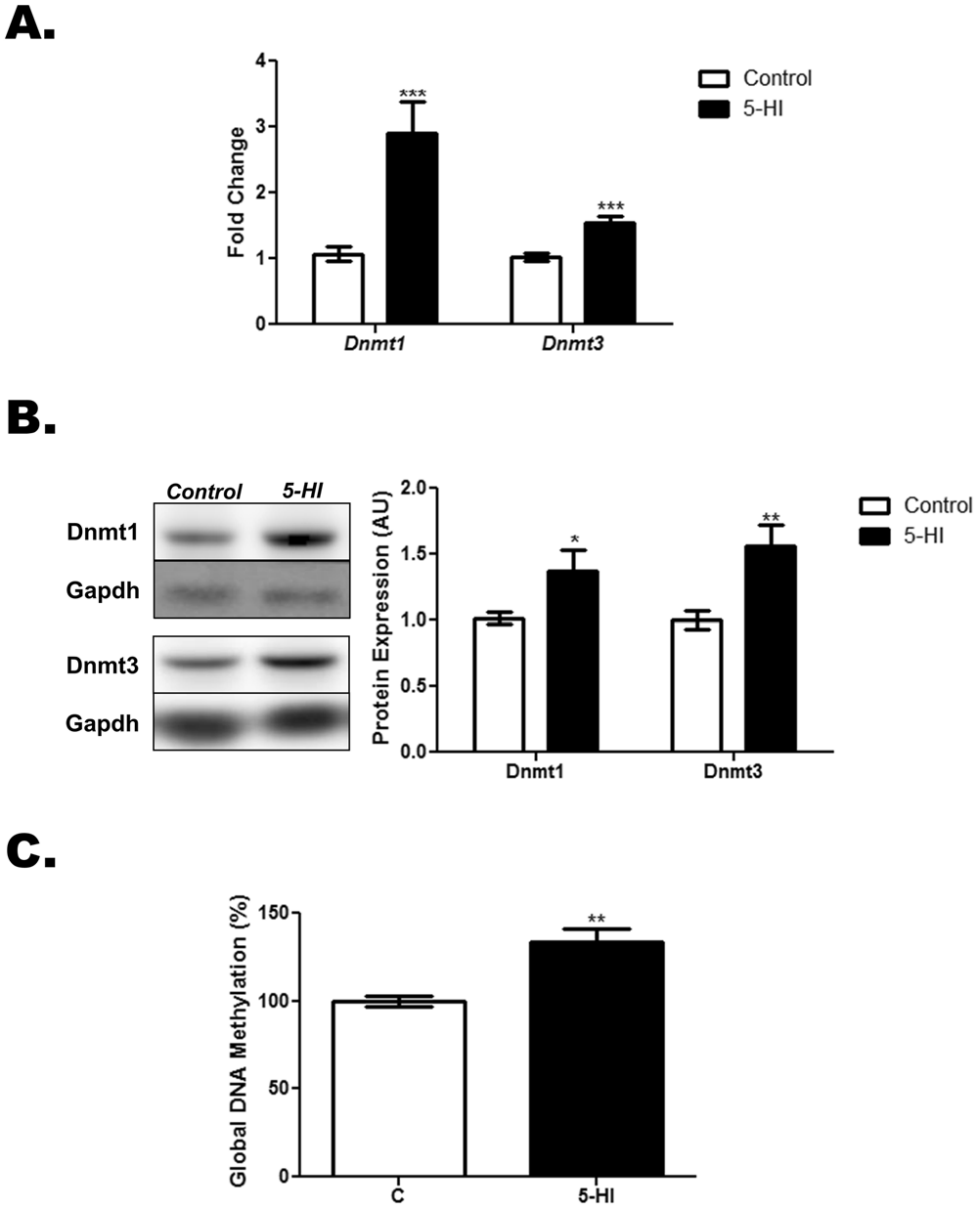
Maternal IE exposure increases Dnmts expression and DNA methylation in the thyroid of the adult male offspring. Thyroid lobes were obtained from the adult male offspring of control (C) or IE-exposed rat dams (5-HI). (**A**) *Dnmt1* and *Dnmt3* mRNA content were analyzed by Real-Time PCR and normalized to *Rpl19* mRNA content (n = 10/group). (**B**) Total Dnmt1 and Dnmt3 protein content were analyzed in the thyroid through Western blotting, using Gapdh as loading control (n = 10/group). Representative western blots are shown in the left panel. (**C**) Thyroid DNA methylation was assessed through Imprint® Methylated DNA Quantification Kit. Results are expressed as means ± SEM as fold change, arbitrary units (AU) or percentage (%), respectively. *P < 0.05, **P < 0.01; ***P < 0.001 vs. C (*Unpaired two tailed Student’s t-test*).

Maternal exposure to IE has increased both the mRNA (Fig. 6A) and protein (Fig. 6B) expression of the DNA methyltransferases 1 and 3 in the thyroid of the adult male offspring. Accordingly, global DNA methylation was also significantly increased in the thyroid of these animals in comparison to the control group (Fig. 6C).

### Histones post-translational modifications in the thyroid of IE-exposed adult male offspring

**Figure 7:**
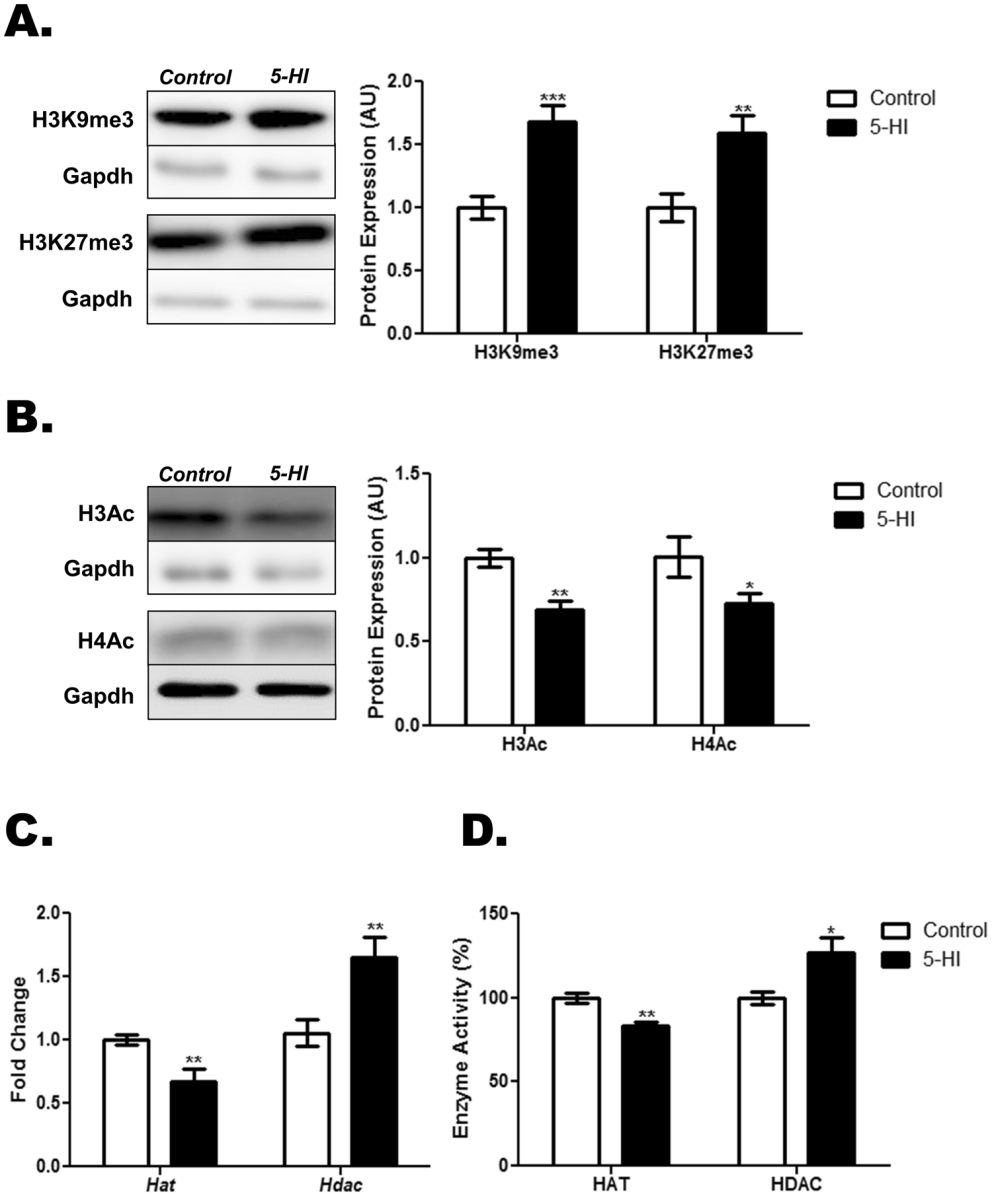
Maternal IE exposure induces post-translational modifications in the thyroid of the adult male offspring. Thyroid lobes were obtained from the adult male offspring of control (C) or IE-exposed rat dams (5-HI). Thereafter, the methylation status of the lysines 9 and 27 of histone H3 (**A**) and acetylation status of the histones H3 and H4 (**B**) were evaluated through Western blotting analysis, using Gapdh as loading control (n = 8/group). Representative western blots are shown in the left panels. (**C**) Thyroid *Hat* and *Hdac* mRNA expression were analyzed by Real-Time PCR and normalized to *Rpl19* mRNA content (n = 10/group). (**D**) Thyroid HAT activity was measured with a colorimetric assay kit, while HDAC activity was assessed with a fluorimetric assay kit. Results are expressed as means ± SEM as arbitrary units (AU) (A-B), fold change (C) or percentage (%) (D),. *P < 0.05, **P < 0.01; ***P < 0.001 vs. C (*Unpaired two tailed Student’s t-test*).

As shown in Fig. 7A, the exposure of rat dams to IE during pregnancy and lactation has increased the lysine-9 and lysine-27 methylation of the histone H3 in the thyroid of adult male offspring. Conversely, IE treatment has reduced the acetylation of the histones H3 and H4 in the thyroid of these animals in comparison to the control ones (Fig. 7B). Moreover, IE-exposed adult male offspring presented a significant reduction of the *Hat* mRNA content and an increased expression of the *Hdac* mRNA in the thyroid gland (Fig. 7C). Furthermore, the HAT activity was reduced, whereas the HDAC activity was increased in the thyroid of the IE-exposed male offspring in comparison to the control animals (Fig. 7D).

## Discussion

The results presented here demonstrate that the exposure of rat dams to IE during pregnancy and lactation induces primary hypothyroidism in their male offspring in adulthood. Additionally, the data strongly suggest that several epigenetic mechanisms are involved in the repression of thyroid gene expression observed in the IE-exposed animals.

Epidemiological data and animal studies suggest that maternal environment during intrauterine and/or lactation periods has a critical role in the programming of gene expression of the offspring^18,19^. Moreover, disturbances during these critical periods of development are commonly related to increased susceptibility to develop neuronal, cardiovascular, endocrine or metabolic diseases in the adult life^20,21^. In accordance, our data indicated that the maternal ingestion of high concentrations of iodine significantly altered the function of the hypothalamus-pituitary-thyroid axis of the male rat offspring in adulthood. This finding was in agreement with earlier epidemiological observations that reported the occurrence of hypothyroidism and goitre in the newborns of IE-exposed mothers^12,22,23^. However, our study was the first to evaluate, using an animal model, the impact of maternal IE exposure in the function of hypothalamus-pituitary-thyroid axis of the male offspring during adult life.

The hypothyroid condition was confirmed by several molecular and biochemical parameters. Firstly, there was a significant increase of serum TSH levels in the offspring of IE-exposed rat dams. This result seems to be associated with the reduction of serum T_3_ and T_4_ concentrations also observed in these animals. Indeed, since *Tsh* mRNA expression is down regulated by TH^24^, the increased mRNA expression of both subunits of *Tsh* in the pituitary reinforce the hypothyroid condition in the IE-exposed offspring. Additionally, the expression of other genes that are known to be regulated by TH, as *Dio2* and *Gh* ^25–27^ were also altered in the pituitary of the offspring derived from IE-treated rat dams. Importantly, the reduced action of TH in the male offspring’s hypothalamus-pituitary axis was also confirmed by the increased expression of *Trh* and *Trhr* mRNAs that are known to be down regulated by T_3_^28^. In fact, the latter result also suggests an increased action of Trh in the thyrotropes of IE-exposed animals and could justify the increased secretion rate of TSH in the bloodstream of these animals.

The reduced TH action in peripheral tissues was strengthened by the decreased DHW and DHW/BW ratio in the offspring of IE-exposed rat dams. As described before, TH regulate the expression of several structural and functional proteins in the heart^29^. Therefore, the reduction of DHW is commonly observed in hypothyroid animals^30^.

The repression of thyroid gene expression and, consequently, the reduced serum TH levels in IE-exposed animals may be related to the significant reduction of the mRNA/protein expression of  Pax8 and Nkx2.1. In fact,  these transcription factors are responsible for controlling the expression of several differentiation genes in the thyroid cells^31^. In addition, it has been reported that the oxidative stress reduces the transcriptional activity of these transcription factors and their binding to the promoters of thyroid genes^32^. Moreover, previous studies demonstrated that IE acutely increases the production of reactive oxygen species (ROS) in the thyroid^33^. Indeed, as demonstrated here, maternal exposure to IE during pregnancy and lactation augmented the content of carbonylated proteins in the thyroid of the adult male offspring, suggesting an increased oxidative stress in this gland. Although the protein carbonylation is an important hallmark of oxidative damage^34^, the absence of additional analyses about other oxidative stress parameters is a limitation of this study.

Taken together, our data suggest that the IE-induced reduction of Pax8/Nkx2.1 content associated with their reduced transcriptional activity might contribute to the decreased expression of genes/proteins involved in the TH synthesis and secretion (Tshr, Nis, Tpo, Tg and Mct8). Interestingly, the reduction of the thyroid follicles’ diameter and the decreased colloid content within the follicles in the IE-exposed animals concur with the observed reduction of Tg protein content in these animals. It is worth noting that the physiological mechanism that protects the thyroid gland from the deleterious effects of IE, known as the Wolff-Chaikoff effect, is not mature until the 36th week of pregnancy in humans ^35^. Therefore, the impact of a direct effect of IE on the regulation of thyroid gene expression during the intrauterine period is probably more intense in the developing foetus than in the adult individuals.

Besides the impairment of the thyroid function in the IE-exposed offspring, the decreased serum TH levels, especially T_3_, might also be a consequence of the reduced activity of D1 in the liver and the kidney. In fact, it is well known that D1 activity is the main responsible for the peripheral conversion of T_4_ to T_3_^36^. Furthermore, the impaired D1 activity is in accordance with the observed reduction of *Dio1* mRNA in both tissues of IE-exposed animals.

The adult male offspring of the rat dams exposed to IE during pregnancy and lactation presented an increased expression of Dnmt1 and Dnmt3 in the thyroid. Actually, both enzymes are involved in the establishment and maintaining of cytosine methylation in the DNA^37^. Therefore, this result is in accordance with the increased global DNA methylation status that was observed in the thyroid of the IE-exposed offspring rats. Moreover, these data concur with the repression of thyroid gene expression in these animals, since DNA methylation is intimately related to the inactivation of gene transcription^38^. The trigger event responsible for the increased DNA methylation in IE-exposed animals need to be further explored. Indeed, previous studies reported that the hypothyroid condition increased the DNA methylation and the expression of Dnmts in the hippocampus and muscle of adult rats^39,40^. Therefore, the decreased TH circulating levels in the IE-exposed animals could be involved in the increased DNA methylation of the thyroid gland. However, a direct role of IE in this parameter cannot be ruled out. Finally, the increased oxidative stress in the thyroid gland that was reported here might also be involved in the increased DNA methylation, since ROS have been previously associated with several molecular responses that affect the cellular epigenome^41^.

Additionally, post-translational modifications in the N-terminal tails of histones are also involved in the activation or repression of gene transcription^42^. In accordance, our data indicated that the thyroid of IE-exposed rats presented hypermethylation in the lysines 9 and 27 of the histone H3 as well as reduced acetylation of the histones H3 and H4. The latter results are in agreement with the reduced expression/activity of HAT and increased expression/activity of HDAC in the thyroid of IE-exposed animals. It is noteworthy that the methylation of histones is commonly associated with transcriptional repression, whereas the acetylation of the N-terminal tail of these proteins is usually associated with transcriptional activation^43^. As discussed above for DNA methylation, hypothyroidism has also been previously associated with reduced acetylation of histones in the hippocampus as well as with increased activity of histones deacetylases in the liver^39,44^. Moreover, a previous study indicated that TH regulate the myosin expression in the cardiac cells through post-translational modifications in the histones^45^. Therefore, the hypothyroid condition observed in the IE-exposed animals could also be associated with the histones post-translational modifications that were described herein. Even so, a direct role of IE on this molecular event cannot be excluded.

It is worth mentioning that the gene expression programming during the intrauterine life has been described as a “predictive adaptive response” that promotes a physiological adjustment in the offspring to guarantee their survival in the same environment of their mother^46^. Therefore, the IE-induced reduction of thyroid gene/protein expression could be an adaptive mechanism to protect the offspring’s thyroid from the deleterious effects triggered by IE and to guarantee the TH production if these animals were maintained in an IE environment. However, since the IE-exposed animals were supplied with a normal iodine diet after the end of the weaning until adulthood, the reduced thyroid gene expression could not be sufficient to guarantee an adequate production of TH, inducing the primary hypothyroid condition that was observed in these animals in the adult life. Importantly, future studies about the thyroid function of the IE-exposed offspring in the end of the weaning (PND21) will add valuable information about the predictive adaptive response hypothesis.

In summary, our results demonstrated that the exposure of rat dams to IE altered the hypothalamus-pituitary-thyroid axis function of their male offspring in adult life. Additionally, our data also suggest that increased DNA methylation and histones post-translational modifications are involved in the repression of thyroid gene expression in the IE-exposed animals. Therefore, by using an animal model, our data reinforce the pivotal role of maternal diet in the foetal development and the early programming of adult diseases.

## Methods

### Animals and Treatments

Virgin male and female Wistar rats were obtained from the Animal Breeding Centre at the Institute of Biomedical Sciences, University of Sao Paulo. The animals were housed at constant temperature (23 ± 1 °C), 12:12-h light-dark cycle schedule, with free access to food and water. At eight weeks of age, female Wistar rats were mated with male rats at a proportion of 2:1. After confirming the pregnancy through the presence of spermatozoa in the vaginal smear, the pregnant rats were housed in individual cages and randomly divided into the following groups: *Control (C):* Rat dams supplied with distilled water during pregnancy and lactation periods. *5-HI:* Rat dams supplied with distilled water supplemented with 0.6 mg/L NaI during pregnancy and lactation periods. The dose of treatment was chosen based on previous studies and represents five times the normal daily consumption of iodine by rats^9,47^.

**Figure 8:**
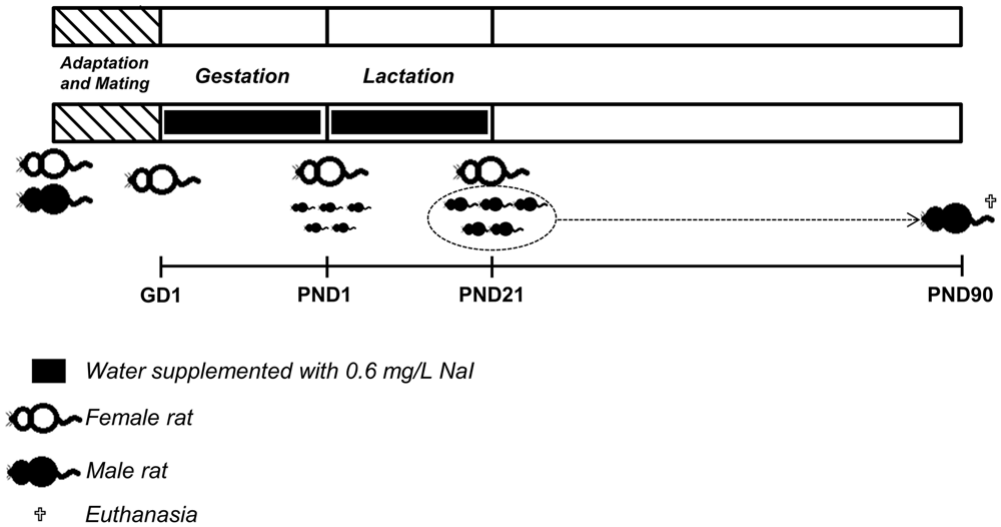
Schematic representation of experimental protocol. Male and female Wistar rats were mated and after confirming the pregnancy through the presence of spermatozoa in the vaginal smear, the pregnant rats (GD1) were treated or not with water supplemented with 0.6 mg/L NaI during pregnancy and lactation periods. At postnatal day (PND) 1, the litters were culled to eight pups per dam and kept at this proportion until the end of the weaning. At PND21 the male rats were housed in cages and maintained with water and food *ad libitum*. At PND90, the adult male rats were anesthetized and euthanized by decapitation and tissues were collected for performing several molecular and biochemical analyses.

At postnatal day (PND) 1, the litters were culled to eight pups per dam and kept at this proportion until the end of the weaning. At PND21 the male rats were housed in cages (five animals per cage) and maintained with water and food *ad libitum*. At PND90, the rats were anesthetized and euthanized by decapitation (Fig. 8). The blood, hypothalamus, pituitary, thyroid gland, heart, kidney and liver were collected for performing the molecular and/or biochemical analysis described below.

The experimental protocol was approved by the Institute of Biomedical Sciences/University of São Paulo-Ethical Committee for Animal Research (no. 155/2012) and the protocols are in accordance with the ethics principles in animal research adopted by the National Council for the Control of Animal Experimentation.

### Gene expression analysis

Total RNA was extracted using Trizol following the manufacturer’s protocol (LifeTechnologies, Carlsbad, CA, USA). Then, the mRNA expression of thyrotropin releasing hormone (*Trh*), type 2 iodothyronine deiodinase *(Dio2)*, thyrotropin releasing hormone receptor (*Trhr*), alpha subunit of thyrotropin *(Tsha)*, beta subunit of thyrotropin *(Tshb)*, growth hormone (*Gh*), paired box 8 *(Pax8)*, thyroid transcription factor 1 *(Nkx2.1)*, thyrotropin receptor *(Tshr)*, sodium-iodide symporter *(Slc5a5)*, thyroperoxidase *(Tpo)*, thyroglobulin (*Tg*), monocarboxilate transporter 8 *(Mct8)*, type 1 iodothyronine deiodinase *(Dio1)*, DNA methyltransferase 1 *(Dnmt1)*, DNA methyltransferase 3 *(Dnmt*3*)*, histone acetyltransferase *(Hat)* histone deacetylase *(Hdac)* were evaluated by RT-qPCR according to the manufacturer’s recommendations (Invitrogen, Carlsbad, CA, USA). Primer sequences are described in Supplementary Table 1. The 2^−ΔΔCt^ method was used to measure gene expression variations using *Rpl19* as a housekeeping gene. Melting curves were analysed to confim the amplification of a single PCR product.

### Western blotting analysis

Pituitary and thyroid total protein content were extracted using RIPA buffer [50 mM Tris (pH 8), 150 mM NaCl, 1% Nonidet P-40, 0.5% sodium deoxycholate, 0.1% SDS] supplemented with protease inhibitor cocktail. Therefore, the total proteins extracts were subjected to electrophoresis in 6, 10, or 15% polyacrylamide gels and transferred to nitrocellulose membranes, which were blocked and incubated with specific primary and secondary antibodies diluted in 3% BSA, 0.1% Tween 20, Tris-buffered saline overnight at 4 C. After chemiluminescent reaction detection, blots densitometries were analysed by using the ImageJ Software (National Institutes of Health, Bethesda, MD). Primary antibodies are described in Supplementary Table 2.

### Thyroid histological analysis

Thyroid histology was performed as previously described^48^. Hematoxylin-eosin-stained sections were examined in a Nikon Eclipse E600 microscope and photographs were captured with a digital camera (Roper Scientific, Trenton, NJ, USA).

### Thyroid oxidative stress analysis

Protein carbonylation status in the thyroid gland was analysed by using the OxyBlot™ Protein Oxidation Detection Kit, following the instructions of the manufacturer (EMD Millipore Headquarters, Billerica, MA, USA). Briefly, the extracted proteins were subjected to Western Blotting, as described above. Therefore, the membranes were subsequently incubated with a primary antibody to detect the carbonyl groups and then with a peroxidase-conjugated secondary antibody. Blots were developed and analysed as described above. Ponceau S staining was used as a loading control to normalize the analysis of the protein carbonylation content in the thyroid gland. Results are presented as percentage in comparison to the control group.

### D1 activity analysis

Liver and kidney D1 activity were determined as previously described^49^. D1 activity was expressed as picomol rT3.min^−1^.mg^−1^.

### DNA extration and DNA global methylation analysis

Total DNA was extracted with the PureLink® Genomic DNA Kit, following the manufacturer’s intructions (LifeTechnologies, Carlsbad, CA, USA). Thereafter, global DNA methylation status was assessed by the Imprint® Methylated DNA Quantification Kit (Sigma Aldrich, St. Louis, MO, USA).

### Histone post-translational modifications assessment

The analysis of the methylation or acetylation status of histones H3 and H4 N-terminal tails was performed by Western blotting, following the protocol described above. The post-translational modifications were assessed by using specific antibodies to detect histone methylation (H3K27me3 and H3K9me3) or histone acetylation (H3Ac e H4Ac). In addition, the activity of thyroid HAT and HDAC enzymes were evaluated by using a HAT Activity Colorimetric Assay Kit and a HDAC Activity Assay Kit, respectively, following protocol indicated by the manufacturer (Sigma Aldrich, St. Louis, MO, USA).

### Determination of TSH, T_3_, T_4_ serum concentrations

Rat TSH, T_4_, and T_3_ serum levels were determined by the Milliplex Luminex® kit #RTHY-30K (EMD Millipore Headquarters, Billerica, MA, USA), following the manufacturer’s instructions.

### Data analysis

All data was reported as means ± SEM. Comparisons between two groups were made using the Unpaired two-tailed Student’s t test. Statistical analysis was performed by using GraphPad Prism (GraphPad Software, San Diego, CA). Differences were considered statistically significant at p-value < 0.05.

## Electronic supplementary material

Supplementary Material
