# Supplementary material for: Maternal Exposure to Iodine Excess Throughout Pregnancy and Lactation Induces Hypothyroidism in Adult Male Rat Offspring
Source: Sci Rep. 2017 Nov 15;7:15591. doi: 10.1038/s41598-017-15529-9 (PMC5688151; doi:10.1038/s41598-017-15529-9)
Supplement: Supplementary file 1 — Supplementary Material [file 41598_2017_15529_MOESM1_ESM.docx]

**Supplementary Material – "*Maternal Exposure to Iodine Excess Throughout Pregnancy and Lactation Induces Hypothyroidism in Adult Male Rat Offspring"***

Caroline Serrano-Nascimento^1*^, Rafael Barrera Salgueiro^1^, Thiago Pantaleão^2^, Vânia Maria Corrêa da Costa^2^, Maria Tereza Nunes^1^

^1^Department of Physiology and Biophysics, Institute of Biomedical Sciences, University of São Paulo, São Paulo, Brazil.

^2^ Carlos Chagas Filho Biophysics Institute, Federal University of Rio de Janeiro, Rio de Janeiro, Brazil.

*carolsn.83@gmail.com

**Supplementary Table 1 –** Primers used for gene expression analysis through Real-Time PCR.

|  | Forward | Reverse |
| --- | --- | --- |
| *Trh* | CGGTGCTGCCTTAGACTCCTGGA | GCCGGGGTGCTGTCGTTTGT |
| *Tshb* | GGCAAACTGTTTCTTCCCAA | GTTGGTTTTGACAGCCTCGT |
| *Tsha* | CACTCTGGCATTTCCCATTA | GCCAGGTCCAAGAAGACAAT |
| *Trhr* | TGGCCACTGTGCTTTACGGG | CAACCACTGCAAGCATCTTGG |
| *Dio2* | GGACCGATGTGCTGCAGCCC | GGCGTGAGCTTCTTCAATGTA |
| *Gh* | TCAAGAAGGACCTGCACAAG | GTGGCAGTTGCCAGAGTACA |
| *Pax8* | CTGTCTCAGGCCAAGTC | CAGCCTGCTGAGTTCTCCAT |
| *Nkx2.1* | ACCTTACCAGGACACCATGC | TTCTTGCTCACGTCCCCCAG |
| *Slc5a5* | AGCCTCGCTCAGAACCATTC | GTGTACCGGCTCCGAGGAT |
| *Tshr* | GGCTGCTGGCTGCTTCTTTT | TCAGACGCATGATCAAAATGAAA |
| *Tg* | CTCAGGACGATGGGCTTATCA | GTTCGGCCTTGGCTTTCTTC |
| *Tpo* | ACAGTTCTCCACGGATGCACTA | GGCAAGCATCCTGACAGGTT |
| *Mct8* | AGCCTGCGCTACTTCACCTA | GGCCAGCTTGATTCTGTCTC |
| *Dio1* | ATTTGACCAGTTCAAGAGACTCG | GGCGTGAGCTTCTTCAATGTA |
| *Dnmt1* | GGTGTTGTCTACCGACTGGG | GGTAATGCTCGGGGTACAGG |
| *Dnmt3* | CACCTTTCCAGATTCGGGAGT | GAGGGAGCATCCTTCGTGTC |
| *Hat* | TACGCTCTTTGCGACCGTAG | GGCCCTGACCTTGAAATGGA |
| *Hdac* | TCAGCCCCACCAATATGCAG | AGCCAGAGGCCTCAAACTTC |
| *Rpl19* | CCAATGAAACCAACGAAATCG | TCAGGCCATCTTTGATCAGCTT |

**Supplementary Table 2 –** Primary antibodies list.

| Peptide/Protein Target | Antibody Name | Manufacter and catalog | Species raised in; monoclonal or polyclonal | Dilution |
| --- | --- | --- | --- | --- |
| TSHB | NIDDK-anti-rat bTSH-IC-1 | NHPP reagents | Rabbit; polyclonal | 1 3000 |
| TSHA | Anti-rat glycoprotein hormone alpha subunit | NHPP reagents | Rabbit; polyclonal | 1 3000 |
| NIS | Anti-rNIS | Dr. Nancy Carrasco | Rabbit; polyclonal | 1 3000 |
| TPO | Anti-Thyroperoxidase (MoAb47) | Santa Cruz  (sc-58432) | Mouse; monoclonal | 1 1000 |
| TSHR | Anti-TSHR antibody | Sigma Aldrich (SAB2102588) | Rabbit; polyclonal | 1 1000 |
| TG | Anti-Thyroglobulin antibody | Abcam  (ab80783) | Mouse; monoclonal | 1 5000 |
| MCT8 | Anti-MOT8 antibody | Abcam  (ab136980) | Rabbit;  policlonal | 1 1000 |
| PAX8 | Anti-Pax8 antibody | Santa Cruz  (sc-81353) | Mouse;  monoclonal | 1 500 |
| NKX2.1 | Anti-TTF1 antibody | Santa Cruz  (sc-13040) | Rabbit; polyclonal | 1 5000 |
| DNMT1 | DNMT1antibody | Cell Signalling  (D59A4) | Rabbit; monoclonal | 1 1000 |
| DNMT3 | DNMT3 antibody | Cell Signalling  (D59A4) | Rabbit; polyclonal | 1 1000 |
| H3K9me3 | Anti-trimethyl-Histone H3 (Lys9) antibody | EMD Millipore  (# 07-442) | Rabbit; polyclonal | 1 1000 |
| H3K27me3 | Anti-trimethyl-Histone H3 (Lys27) antibody | EMD Millipore  (#05-1951) | Mouse; monoclonal | 1 1000 |
| H3Ac | Anti-acetyl-Histone H3 antibody | EMD Millipore  (#06-599) | Rabbit; polyclonal | 1 1000 |
| H4Ac | Anti-acetyl-Histone H4 antibody | EMD Millipore  (#06-866) | Rabbit; polyclonal | 1 1000 |
| GAPDH | Anti-GAPDH antibody | Santa Cruz  (sc-32233) | Mouse; monoclonal | 1 1000 |

**Abbreviations: *THSB***, thyrotropin beta subunit; ***TSHA***, thyrotropin alpha subunit; ***NIS****,* sodium iodide symporter; ***TPO****,* thyroid peroxidase; ***TSHR***, thyrotropin receptor; ***TG***, thyroglobulin; ***MCT8*** monocarboxilate transporter 8; ***PAX8***, paired box gene 8; ***NKX2.1****,* thyroid transcription factor 1; ***DNMT1****,* DNA methyltransferase 1; ***DNMT3****,* DNA methyltransferase 3; ***H3K9me3***, trimethyl-Histone H3 (Lys9); ***H3K27me3***, trimethyl-Histone H3 (Lys27); ***H3Ac***, acetyl-Histone H3; ***H4Ac****,* acetyl-Histone H4; ***GAPDH***, glyceraldehyde 3-phosphate dehydrogenase.

**Supplementary Figure 1**


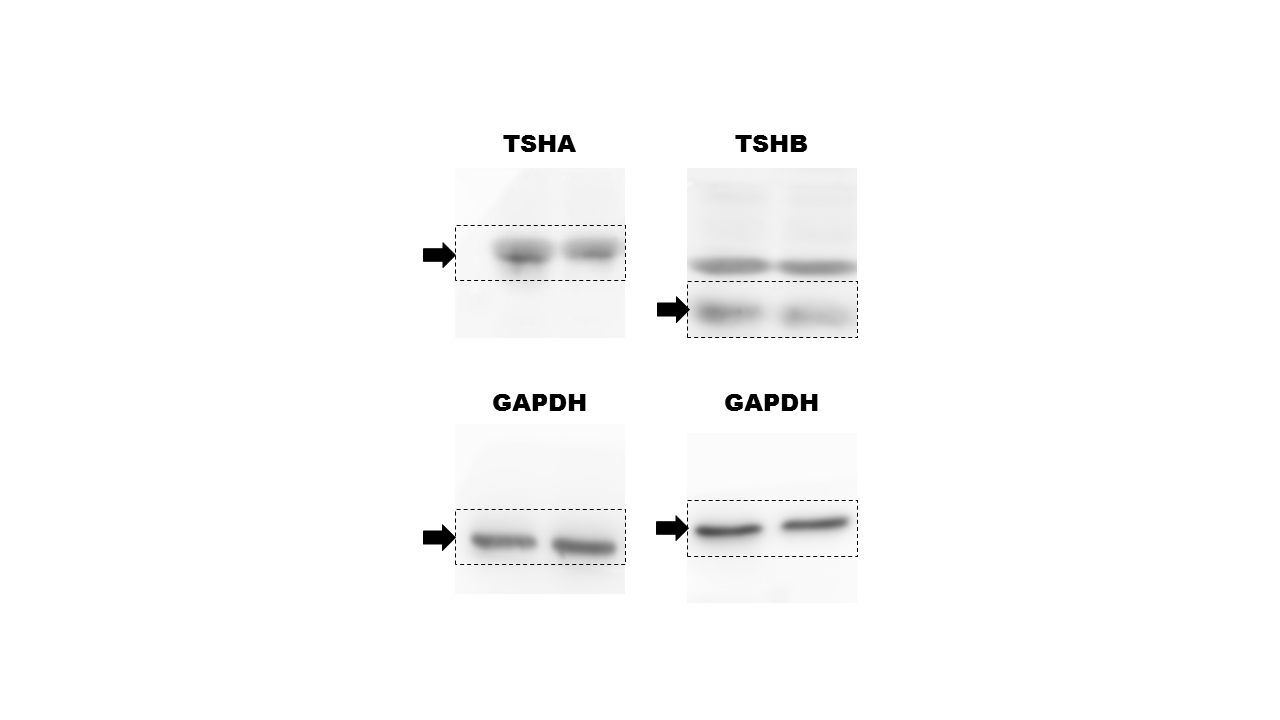


**Supplementary Figure 1 -** **Full-length images of the immunoblots presented in the Figure 1.** Black dot line boxes indicate the cropped images used in Figure 1.

**Supplementary Figure 2**

**Supplementary Figure 2 – Full-length images of the immunoblots presented in the Figure 3.** Black dotted line boxes indicate the cropped images used in Figure 3.


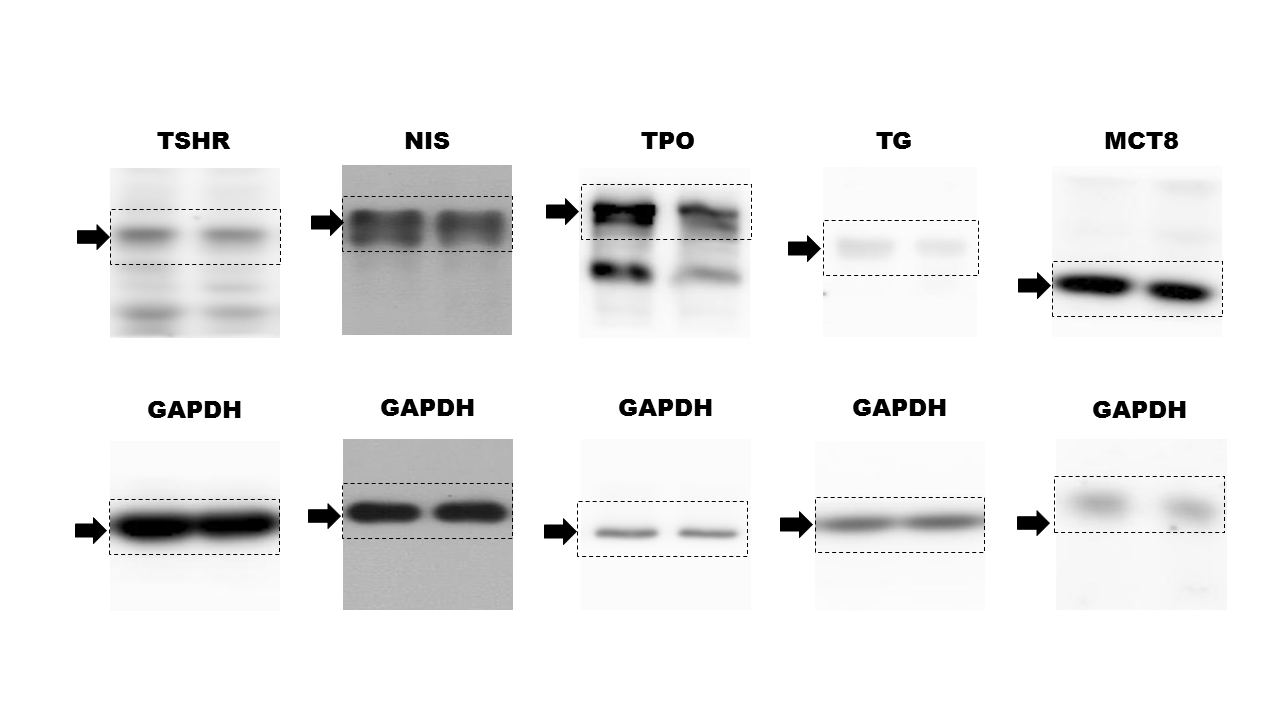


**Supplementary Figure 3**


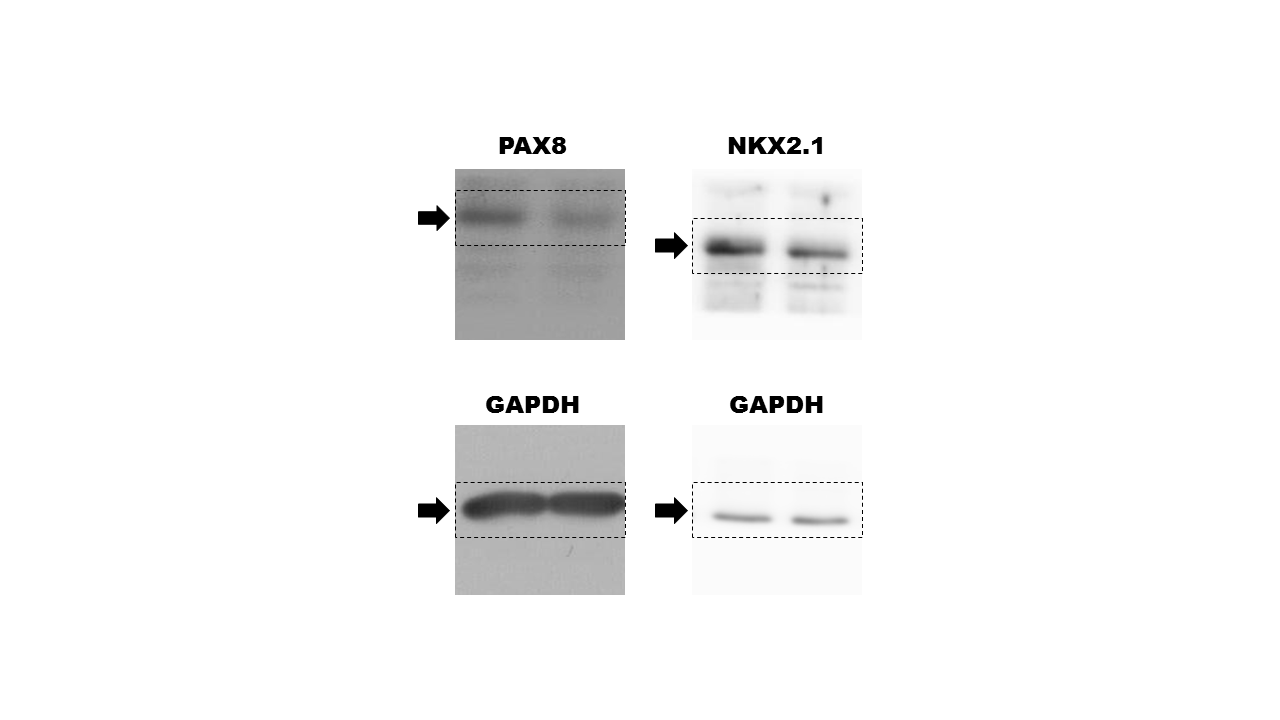


**Supplementary Figure 3 -** **Full-length images of the immunoblots presented in the Figure 4.** Black dotted line boxes indicate the cropped images used in Figure 4.

**Supplementary Figure 4**


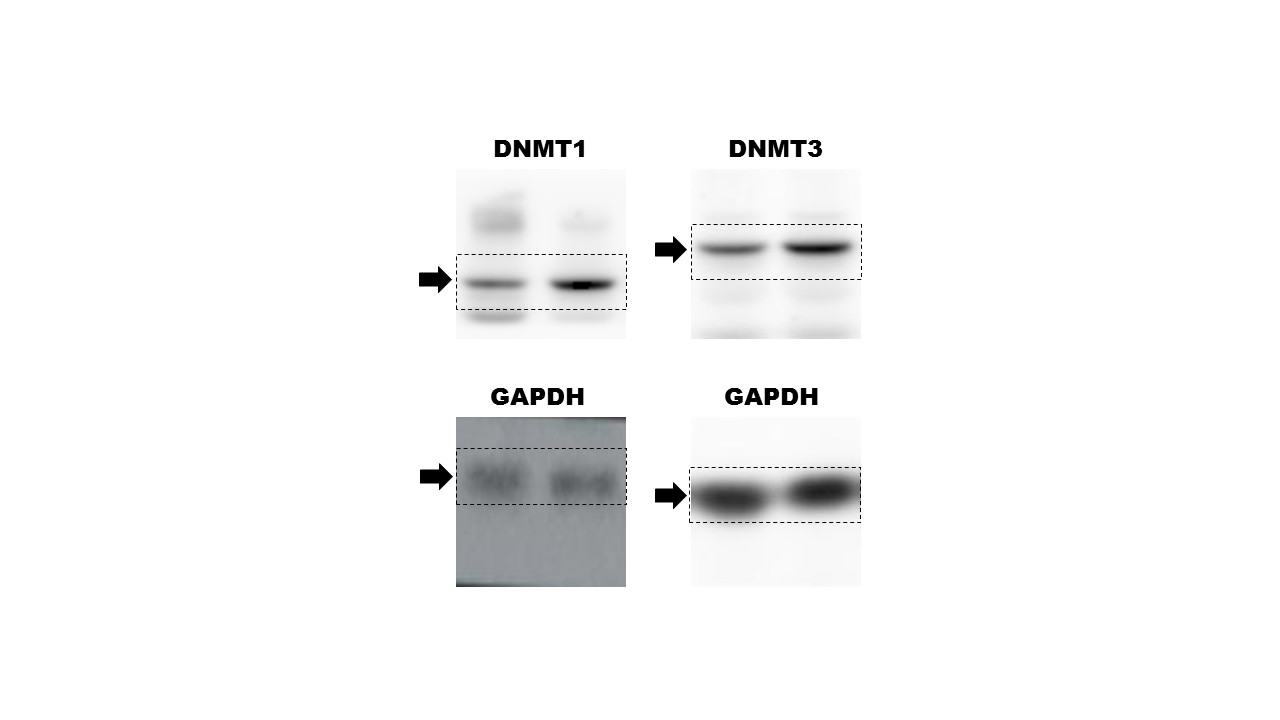


**Supplementary Figure 4 -** **Full-length images of the immunoblots presented in the Figure 6.** Black dotted line boxes indicate the cropped images used in Figure 6.

**Supplementary Figure 5**


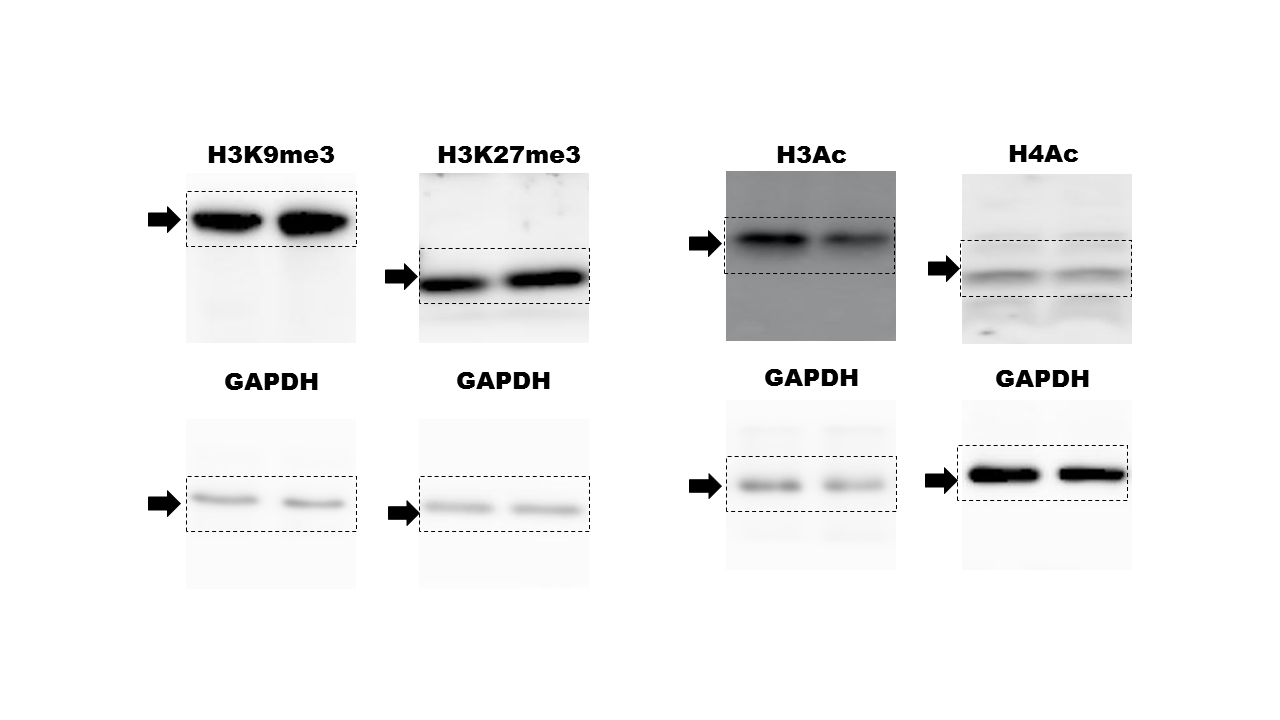


**Supplementary Figure 5 -** **Full-length images of the immunoblots presented in the Figure 7.** Black dotted line boxes indicate the cropped images used in Figure 7.
